# Mental Health and Well-Being in Children

**DOI:** 10.3390/children9081212

**Published:** 2022-08-11

**Authors:** Cristina Nunes

**Affiliations:** Psychology Research Centre (CIP-UAL), University of Algarve, 8005-139 Faro, Portugal; csnunes@ualg.pt

Mental disorders are the largest cause of the burden of disease in the world [1]. Evidence is accumulating on the broad impact of the well-being experienced during childhood and adolescence on physical and mental health across the course of a lifetime. In fact, most of the disease burden affecting adults has its onset during childhood and adolescence [1,2].

There has been a growing concern about the mental health and well-being of children, with increasing demand for counseling services and referrals to mental health services [2]. It is well-established in the existing literature that children and young people who experience positive support from parents and teachers may develop psychological resilience [3,4,5]. Children and adolescents with higher levels of psychological well-being have higher levels of academic achievement, along with higher engagement in school life and satisfaction in their later life, and they are more productive workers [4,5,6].

Family factors, including the quality of parental care, can make a massive difference to the early life pathways of children, for better or worse [6,7]. Understanding how best to intervene to support parents is a key challenge. Thus, there is a strong need to expand our knowledge on how to reduce risk factors and promote protective environments.

This Special Issue addresses this topic with 13 papers written by different researchers from different countries, sharing findings, perspectives, and approaches, to promote child mental health and well-being. The common feature among them is the objective to enhance knowledge about mental health and well-being in children.

Regarding the role of the family in the mental health and well-being of children, we should emphasize the research produced by Baena et al. [8] and Carmo et al. [9] in which they studied, respectively, the role of parenting alliance and the parental perfectionism and parenting styles in children adjustment.

Studying the significant role of schools, Ramberg [10] and Gómez-Baya [11] contribute to this Special Issue by highlighting how the school environment can impact the well-being of children, their mental health, and their families.

In a very specific topic, children followed by child protective services, the contributions of Lemos et al. [12], Pires et al. [13], Salomão et al. [14], and Stuart et al. [15] present different orientations on how to intervene with these families in children at risk situations.

Nunes et al. [16] extend this discussion by presenting a program of online parenting support on children’s quality of life, providing insight into its impact on parents and children.

Faísca et al. [17], Gomes et al. [18], Pechorro et al. [19], and Spoto et al. [20] provide the results of their research on the validation and adaptation of instruments (Lab-TAB, Quiet Time Program, SUPPS-P Impulsive Behavior Scale, and the Multidimensional State Boredom Scale, respectively), which have practical implications in the assessments and interventions involving children and adolescents.

As the Guest Editor of this Special Issue, I would like to thank all authors for their insightful contributions, and all people associated with this publication at the MDPI Editorial Office of *Children*, in particular Dr. Sari A. Acra, Editor in Chief, who had supported this Special Issue of *Children*, as well as Kelly Qiao and Dorothy Zhang, Assistant Editors. Special thanks to all academic editors and peer reviewers who contributed a significant amount of time and made constructive comments.
